# The role of lipocalin 2 in brain injury and recovery after ischemic and hemorrhagic stroke

**DOI:** 10.3389/fnmol.2022.930526

**Published:** 2022-09-15

**Authors:** Jingwei Zhang, Zeyu Wang, Hao Zhang, Shuwang Li, Jing Li, Hongwei Liu, Quan Cheng

**Affiliations:** ^1^Department of Neurosurgery, Xiangya Hospital, Central South University, Changsha, China; ^2^National Clinical Research Center for Geriatric Disorders, Changsha, China; ^3^Department of Rehabilitation, The Second Xiangya Hospital, Central South University, Changsha, China; ^4^Clinical Diagnosis and Therapy Center for Glioma of Xiangya Hospital, Central South University, Changsha, China; ^5^Department of Clinical Pharmacology, Xiangya Hospital, Central South University, Changsha, China

**Keywords:** LCN2, neuroinflammation, ischemic stroke, intracerebral hemorrhage, intraventricular hemorrhage, subarachnoid hemorrhage

## Abstract

Ischemic and hemorrhagic stroke (including intracerebral hemorrhage, intraventricular hemorrhage, and subarachnoid hemorrhage) is the dominating cause of disability and death worldwide. Neuroinflammation, blood–brain barrier (BBB) disruption, neuronal death are the main pathological progress, which eventually causes brain injury. Increasing evidence indicated that lipocalin 2 (LCN2), a 25k-Da acute phase protein from the lipocalin superfamily, significantly increased immediately after the stroke and played a vital role in these events. Meanwhile, there exists a close relationship between LCN2 levels and the worse clinical outcome of patients with stroke. Further research revealed that LCN2 elimination is associated with reduced immune infiltrates, infarct volume, brain edema, BBB leakage, neuronal death, and neurological deficits. However, some studies revealed that LCN2 might also act as a beneficial factor in ischemic stroke. Nevertheless, the specific mechanism of LCN2 and its primary receptors (24p3R and megalin) involving in brain injury remains unclear. Therefore, it is necessary to investigate the mechanism of LCN2 induced brain damage after stroke. This review focuses on the role of LCN2 and its receptors in brain injury and aiming to find out possible therapeutic targets to reduce brain damage following stroke.

## Introduction

Stroke has become a global burden, causing death and long-term disability in young adults (Smajlovic, 2015). Reliable evidence suggests that stroke will remain the world’s second leading cause of death after cardiovascular disease until 2030 (Mathers and Loncar, 2006; Ovbiagele et al., 2013). Furthermore, the stroke rate is more than 70% in low- and middle-income countries, and the rate of stroke-related death and disability-adjusted life-years is more than 80% (Johnson et al., 2016; Donkor, 2018). The primary treatments for ischemic stroke include tissue plasminogen activator (tPA) for thrombolysis and mechanical thrombectomy. In addition, reducing the volume of hematoma is the principal treatment goal for hemorrhagic stroke (Sattin and Zivin, 2007; Novakovic et al., 2009; Rymer, 2011). However, the overall treatment outcome of stroke remains unsatisfactory. Therefore, more effective treatments are urgently required to control the disability and mortality of stroke patients.

The lipocalin family consists of many small extracellular proteins, which regulate the transportation of small hydrophobic molecules, including lipids, hormones, and vitamins (Flower, 1996). Increasing evidence indicated that lipocalins play an essential role in various physiological and pathological processes, including innate immune response, iron homeostasis, cellular communication, apoptosis, tumorigenesis, pheromone transport, and retinoid-binding (Flower, 1996; Ghosh et al., 2020). LCN2, also known as neutrophil gelatinase-associated lipocalin (NGAL), p25, and oncogene 24p3, comes from the lipocalin superfamily (Moschen et al., 2017). According to recent research, LCN2 increases rapidly and significantly after stroke and plays a vital role in brain injury (Zhao N. et al., 2019; Wang et al., 2020). LCN2 can injure neurons directly, activate and polarize glial cells, destroy the BBB’s integrity, and induce immune infiltrates, causing long-term neurological deficits. However, the underlying mechanism and signaling pathways of LCN2 in stroke are complicated and ambiguous.

Following a stroke, various cells in the central nervous system (CNS), including endothelial cells, vascular smooth muscle cells (VSMCs), glial cells, and neurons, express LCN2 (Bu et al., 2006; Lee et al., 2015; Ni et al., 2015; Wang et al., 2015b). Increasing evidence indicated that LCN2 is associated with the activation and polarization of astrocytes and microglia/macrophages (Jang et al., 2013b; Lee et al., 2015; Zhao N. et al., 2019). In addition, LCN2 expression in VSMCs relates to homeostasis and migration progress (Chung et al., 2013). Studies found that increased LCN2 in circulation causes BBB dysfunction by affecting tight junction protein expression and upregulating pro-inflammatory cytokines (Mondal et al., 2020). However, the fundamental role of LCN2 in neurons remains complex and ambiguous. Chen X. et al. (2020) found that upregulated LCN2 in the rat hippocampal neurons induced neuronal apoptosis in a mitochondrion-related manner after injury. While in another research, Xing et al. (2014) concluded that LCN2 from injured neurons might send a “help-me” signal that activates glia cells into pro-recovery phenotypes. These findings suggest that further research into the precise role and mechanism of LCN2 after stroke is required.

Iron is essential for maintaining neurons’ excitability and regulating ferroptosis under physiological conditions (Vogalis and Harvey, 2003; Yan and Zhang, 2019). Another essential function of LCN2 is to act as a siderophore-binding protein and isolate free iron, which plays a crucial role in tissue iron homeostasis (Holmes et al., 2005; Borregaard and Cowland, 2006; Bachman et al., 2012). In addition, studies indicated that LCN2 reduces ferroptosis cell death (Meier et al., 2021). However, the role of LCN2 as an iron regulator in stroke is still far from understood.

This review focuses on the mechanisms based on previous studies published online, discusses the role of LCN2 in brain injury after stroke, summarizes the underlying pathological progress, and provides a comprehensive introduction to the clinical intervention-based study. To fully elaborate on this topic, published research from PubMed in clinical and basic studies and review or meta-analysis were included, using the following terms: “Lipocalin 2,” “brain injury,” “receptor,” “stroke,” “intracerebral hemorrhage (ICH),” “intraventricular hemorrhage (IVH),” and “subarachnoid hemorrhage (SAH).” The inclusion criteria state that selected articles must be related to this topic and published in peer-reviewed journals. Articles written in languages other than English are among the exclusion criteria.

## Construction and function of LCN2

Lipocalins are a group of small, monomeric proteins with 100–300 amino acid residues and diameters of approximately 40 A, expressed in prokaryotes and eukaryotes (Goetz et al., 2000). Lipocalins’ three-dimensional (3D) structures are highly unified and conserved, characterized by a central eight-stranded hydrogen-bonded up-and-down β-barrel (Breustedt et al., 2006). An α-helix resting on the β-barrel occupies the carboxy-terminal end. Following the amino-terminal end is a coiled polypeptide segment that is eventually tightened by a disulfide bond. The eight antiparallel strands (named from A to H) in the β-barrel are arranged in a (+1) 7 topology and surround the center in a right-handed manner so that a part of chain A is hydrogen-bonded to chain H again (Schiefner and Skerra, 2015). The bottom of the β-barrel is dominated by short loops and numerous hydrophobic side chains, such as various aromatic residues. In contrast, the top end usually maintains a high affinity for solvents. In addition, four long loops and four pairs of β-strands form an entrance to a pocket-shaped cavity, allowing lipocalins to dwell in various sizes and types of chemical ligands and metabolites, including lipids, steroids, hormones, vitamins, and cofactors. Based on the lengths and amino acid sequences of four loops, these ligands confer specific functions to each lipocalin member.

**FIGURE 1:**
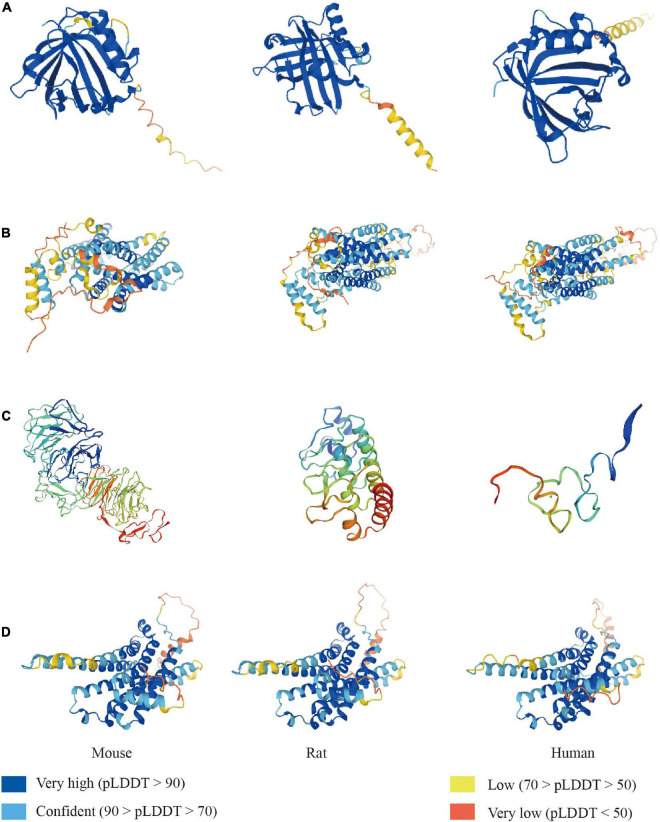
The LCN2 and its receptors fold in mouse, rat, and human. Construction of LCN2 **(A)** and 24p3R **(B)** predicted from the alphafold protein structure database. Construction of megalin **(C)** predicted from the Swiss-model database. Construction of MC4R **(D)** predicted from the alphafold protein structure database.

Similar to lipocalins, LCN2 has a cup-shaped calyx made of eight antiparallel hydrophobic residues, providing binding sites for small lipophilic molecules (Chu et al., 1998; Goetz et al., 2000) (Figure 1A). LCN2 was first detected in the human neutrophil granules as a glycoprotein covalently combined with the 92-kDa gelatinase B (matrix metalloprotein, MMP9). LCN2 has also been described as an acute phase hydrophobic molecular involved in an injury, bacterial infection, inflammation, and iron-related oxidative stress (Kjeldsen et al., 2000; Srinivasan et al., 2012; Jha et al., 2013). Current binding sites for LCN2 ligands are those hydrophobic aromatic and aliphatic residues such as C87, F27, F123, L94, V33, V121, and W31 at the base of β-barrel (Bao et al., 2015). The free SH group of residues C87 at the end of the short loop was found to binding MMP9 and regulated its function. Gram harmful bacteria sequester iron from the host through Enterobacter (Ent), an essential hydrophilic siderophore (Raymond et al., 2003). Goetz et al. (2002) discovered in 2002 that LCN2 could regulate iron metabolism by interacting with ferric Ent and related siderophores. Crystallographic analyses revealed three positively charged residues (K12, K134, and R81) occupying the top open end of the β-barrel bound to the siderophore site. The iron-bound form (holo-LCN2) could release iron and upregulate intracellular iron levels. On the contrary, the iron-free form (apo-LCN2) acts as an intracellular siderophore and transfers iron to the extracellular medium, decreasing intracellular iron concentration (Devireddy et al., 2005).

## LCN2 receptors in the brain

LCN2’s multiple functions are attributed to its primary receptors, such as 24p3R and megalin (Figures 1B,C). 24p3R, also known as neutrophil gelatinase-associated lipocalin receptor (NGALR), brain type organic cation transporter (BOCT), or LCN2R, was coded by human solute carrier family 22-member 17 genes (SCL22A17) (Fang et al., 2007). 24p3R has two forms-24p3R-long (24p3R-L) and 24p3R-short (24p3R-S) and is widely expressed in many tissues, including the brain, spleen, liver, heart, lung, stomach, and kidney (Langelueddecke et al., 2012; Chia et al., 2015). 24p3R protein expresses on neurons and glial cells but not on neutrophils under normal conditions. In the infarct area, neurons, endothelial cells, and astrocytes express 24p3R at the early stage after reperfusion. The study found that 24p3R-L and 24p3R-S expression may vary by gender and developmental stage (De La Chesnaye et al., 2018). The author found that LCN2/24p3R pathway might mediate either cell proliferation in gonadotropin independent or dependent manners. LCN2 mediates iron transportation and cell apoptosis through binding 24p3R.

When LCN2 was not present, 24p3R combined with other extracellular proteins such as toxic cadmium-MT complex (Cd2 + 7-MT), albumin, transferrin, and metallothionein (Devireddy et al., 2001; Langelueddecke et al., 2013, 2014). For example, Dizin et al. (2013) found that LCN2/24p3R signaling pathway regulates the endocytosis of albumin and then promotes the activation of TGF-β1 and NF-κB pathways, which eventually initiates inflammation.

Previous studies demonstrated that many cell types in the CNS express 24p3R after stroke. Toyota et al. (2019) demonstrated that 24p3R positive cells were astrocytes, microglia/macrophage, and oligodendrocytes in white matter after stroke. Neurons and endothelial cells also expressed 24p3R under ischemic conditions. In a brain ischemic/reperfusion model, 24p3R was first colocalized with endothelial cells and astrocytes in the infarct core (Jin et al., 2014b).

Moreover, neurons in the normal sites around the infarct area expressed 24p3R 24 h after reperfusion. According to the findings, LCN2/24p3R on neurons may directly injure neurons and promote neuroinflammation, resulting in brain injury. Elevated LCN2/24p3R on epithelial cells in the choroid plexus contribute to the enlargement of the ventricle after SAH (Marques et al., 2008; Shishido et al., 2016). Wnt/β-catenin regulates the expression of LCN2/24p3R to adapt to changes in the surrounding environment and protects cells from inflammation through TLR-4 (Betten et al., 2018). Runt-related transcription factor (RUNX) proteins, also known as a transcription factor family, regulate stem cell and neuron development and differentiation (Wang and Stifani, 2017; Otalora-Otalora et al., 2019). A study found that the RAS/MAPK pathway meditated 24p3R expression by inducing a switch by combining RUNX3 (an activator of 24p3R *via* binding to 24p3R promoter) to RUNX1 (a repressor of 24p3R *via* recruiting the SIN3a-HDAC corepressor complex).

Megalin, also known as low-density lipoprotein-related protein 2 (LRP2) or gp330, is another crucial receptor of LCN2. It is a 600 kDa protein first cloned and sequenced in 1994, which mediates endocytosis. Megalin contains a signal peptide domino of the amino-terminal, a cytoplasmic tail of the carboxyl-terminal, an extracellular domino, and a transmembrane signal region (Sheng et al., 2009). Megalin express in various tissues such as the brain, lung, eye, intestine, oviduct, uterus, and male reproductive tract and plays a vital role in the development of CNS (Saito et al., 1994). Megalin-deficient mice have abnormal neuroepithelial proliferation, an ordinary ventricular system, and an incomplete olfactory system (Fisher and Howie, 2006). Generally, megalin forms a megalin/cubilin complex to fulfill its role by combining cubilin, a 460 kDa intrinsic factor vitamin B12 receptor protein (Willnow et al., 1996). Other ligands for megalin include insulin, insulin-like growth factor 1 (IGF-1), albumin, MMP9, hemoglobin, vitamin D-binding protein (DBP), retinol-binding protein (RBP), and β2-microglobulin (Cui et al., 1996; Orlando et al., 1998; Christensen et al., 1999; Nykjaer et al., 1999; Gburek et al., 2002). Megalin involves the endocytosis of a wide range of molecules, including LCN2, iron, hemoglobin, albumin, lipoproteins, hormones, toxins, drugs, vitamin-binding proteins, enzymes, immune- and stress-response-related proteins, and so on (Gburek et al., 2002; Nielsen et al., 2016).

Megalin is mainly expressed in epithelial cells and neurons. In normal conditions, the expression of megalin on neurons is essential for neurite outgrowth and synaptic plasticity (Fisher and Howie, 2006; Gomes et al., 2020). Miharada et al. (2008) found that LCN2/megalin also negatively suppressed the growth of erythroid and monocyte/macrophage lineage cells. LCN2/megalin signaling regulates macrophage-epithelial crosstalk and thus plays a vital role in the epithelial cell cycle. LCN2 secreted by macrophages can enhance the expression of epithelial markers by binding to megalin and activating downstream P13K/Akt pathway. This progress is inhibited in LCN2^–/–^ macrophages (Jung et al., 2018). Megalin also mediates the transport of small molecules across the BBB and blood-cerebrospinal fluid (CSF) barrier. IGF-1 is beneficial to the brain because it transports and clears brain amyloid β (Aβ) in the choroid plexus (Kim et al., 2019). Carro et al. (2005) found that megalin can act as a critical inducer that promoted serum IGF-1 cross the choroid plexus barrier to clear Aβ.

Furthermore, a recent study indicated that melanocortin four receptor (MC4R) (Figure 1D) could bind and activate by LCN2 (Dekens et al., 2021). MC4R mediates anorexigenic progress by activating melanocyte-stimulating hormones (MSH). This study discovered that osteoblast-derived LCN2 could cross the BBB and bind to the MC4R expressed in hypothalamic neurons, activating the MC4R-mediated appetite suppression pathway. Further investigation depicted that LCN2 levels in serum are closely related to healthy glucose metabolism index (insulin levels and β-cell function) and body mass index in obsessing mice (Mosialou et al., 2020). LCN2 depletion worsened metabolic parameters and prohibited β-cell function in mouse model β-cell failure. This study provides new directions for studying the role of LCN2 and melanocortins, including adrenocorticotropic hormone (ACTH) and various forms of melanocyte-stimulating hormone (MSH) (Gantz and Fong, 2003). The expression of these three receptors determines the various functions of LCN2 (Table 1). However, research on LCN2/MCR4 is still limited, and more studies are needed to understand its mechanisms fully.

**TABLE 1:** Function and mechanism of LCN2 receptors.

Receptor	Function	Mechanism/Signaling pathway	References
24p3R	Cell proliferation	May mediate cell proliferation through gonadotropin independent or dependent manners	Donkor, 2018
		May mediate cell proliferation through Wnt/β-catenin signaling pathway	Ferdinand and Roffe, 2016
	Iron transportation	24p3R regulates iron uptake depend on the ligand: iron-loaded 24p3 and iron-lacking 24p3	Dixon et al., 2012; Miranda et al., 2019
	Cell apoptosis	Induce apoptosis *via* elevated Bim expression	Dixon et al., 2012; Doll and Conrad, 2017
		IL-3 deprivation promote synthesis and secretion of 24p3 through 24p3R, which induces apoptosis through an autocrine pathway	Du et al., 2021
		Runx3 activates 24p3R to promote apoptosis	Flower, 1996
		LCN2/LCN2R involves in METH-induced mitochondrion-related apoptosis	Chen et al., 2015
		May regulate apoptosis through TLR-4 signaling pathway	Ferdinand and Roffe, 2016
	Protein endocytosis	24p3R mediates albumin endocytosis through NF-κB and TGF-β1 signaling pathways	Egashira et al., 2014
		24p3R mediates protein endocytosis (unknown signaling)	Dyet et al., 2006; Dong et al., 2013; Du et al., 2019
	Whiter matter injury	Induce myelin damage, oligodendrocyte loss (unknown signaling)	Egashira et al., 2015
	Neuronal death	Induce neuronal death (unknown signaling)	Elneihoum et al., 1996; Fang et al., 2007; Feiler et al., 2011
	Ventricular dilation	Enlarge brain ventricle (unknown signaling)	Feiler et al., 2011
	Neuroinflammation	Induce neutrophil infiltration (Erk1/2), pro-inflammatory factors expression and glial activation	Elneihoum et al., 1996; Raymond et al., 2003; Feiler et al., 2011; Rathore et al., 2012; Egashira et al., 2015
	BBB disruption	Increase BBB permeability (unknown signaling)	Elneihoum et al., 1996; Egashira et al., 2015
	Neutrophil ability	MAPK/MEK1/2 signaling pathway	Rymer, 2011
Megalin	Development and cell proliferation	Mediate central nervous system development and cell proliferation (unknown signaling)	Gantz and Fong, 2003; Gu et al., 2009; Guo et al., 2014; Garg et al., 2015
	Regulate cubilin function	Form megalin/cubilin complex to regulate cubilin function	Garton et al., 2016; Gill et al., 2018; Gomes et al., 2020
	Protein endocytosis	Megalin regulate endocytosis of vitamin D, retinol, hemoglobin, insulin, albumin and drugs (unknown signaling)	Goetz et al., 2000, 2002; Gburek et al., 2002; Gill et al., 2018; Ghosh et al., 2020; Gomes et al., 2020
	Cell cycle	Mediate endothelial cell cycle *via* P13K/Akt signaling pathway	Hochmeister et al., 2016
	Transportation of small molecules	Promoted serum IGF-1 cross the choroid plexus barrier	Holland et al., 2018
MC4R	Anorexigenic progress	MC4R mediates anorexigenic progress through activating MSH	Holmes et al., 2005

METH, methamphetamine; MC4R, melanocortin-4-receptor gene; MSH, melanocyte-stimulating hormones.

## Pathological expression of LCN2 in brain after stroke

**FIGURE 2:**
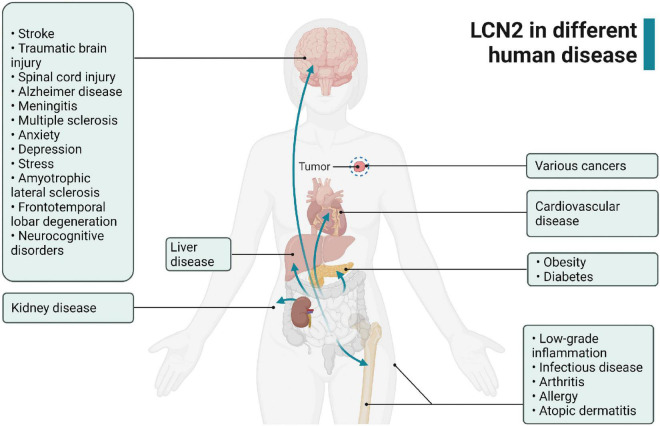
Expression of LCN2 in various human diseases.

LCN2 expresses in numerous organs and tissues under pathological progress, including brain, kidney, liver, heart, blood, tumor tissue, and inflammatory sites (Flo et al., 2004; Naude et al., 2012; Castillo-Rodriguez et al., 2017; Ishii et al., 2017; Marques et al., 2017; Dahl et al., 2018; Chen J. et al., 2020) (Figure 2). Especially, LCN2 elevates significantly in plasma and brain of patients and experimental-induced models after stroke (Table 2). To fully understand the expression landscape of LCN2, we investigated recent research focusing on LCN2 expression and then discussed the possible significant role of LCN2 in the brain after stroke.

**TABLE 2:** Expression of LCN2 in the plasma and brain of stroke patients and stroke-induced animal models.

Type of stroke	Model	LCN2 level	Function/Clinical correlation
Ischemic stroke	tMCAO	↑	LCN2 cause brain injury
			LCN2 released by injured neurons as a help me distress signal that activates microglia and astrocytes into potentially pro-recovery phenotypes.
			LCN2 deficiency attenuates neuroinflammation and brain injury
			LCN2 mainly comes from astrocytes and induce proinflammatory cytokines expression under hypoxic conditions
			LCN2 is an infection-related biomarker to predict clinical outcome in ischemic stroke
	pMCAO	↑	LCN2 null mice did not show any protection
	OGD	↑	LCN2 induce astrocytes activation and polarization
	Carotid artery injury	↑	LCN2 interacts with MMP9 may modulate MMP9 proteolytic activity in the vascular repair process
Hemorrhagic stroke	ICH	↑	LCN2 cause brain injury and up-regulation of ferritin
			LCN2 enhance PRDX2 induced brain injury
			Increased LCN2 may relate to clear iron after ICH
			Thrombin induce the expression of LCN2 through PAR-1 and cause brain injury
	IVH	↑	LCN2 promote M1 polarization in LPS induced brain injury
			LCN2 induce high portality and more ventricular dilation
	SAH	↑	LCN2 induce whiter matter injury after SAH
			LCN2 deletion attenuates MRI and pathological changes in whiter matter after SAH
			LCN2 increases early after SAH and is associated with neuroinflammation and unfavorable outcome
		NA	LCN2 deficiency decreased the number of cerebral thromboembolism

tMCAO, transient middle cerebral artery occlusion; pMCAO, permanent middle cerebral artery occlusion; OGD, oxygen-glucose deprivation; ICH, intracerebral hemorrhage; IVH, intraventricular hemorrhage; SAH, subarachnoid hemorrhage; MMP9, matrix metallopeptidase 9; PRDX2, peroxiredoxin 2; PAR-1, protease activated receptor 1; LPS, lipopolysaccharide; NA, not applicable.

## LCN2 expression in ischemic stroke

Due to the limited interventions, the treatment effect of ischemic stroke remains unsatisfactory (Campbell et al., 2015; Bhaskar et al., 2018). Studies have revealed that early serum and brain LCN2 elevations have become promising biomarkers for patients with ischemic stroke (Elneihoum et al., 1996; Ranjbar Taklimie et al., 2019). Furthermore, elevated LCN2 levels in plasma are associated with poor neurological scores and estimated glomerular filtration rate (eGFR) at 3 months in ischemic stroke patients (Hochmeister et al., 2016). LCN2 upregulated at 6 h in the infarct core and increased at 24 h in the penumbra of the ipsilateral hemisphere under reperfusion conditions. The author found LCN2 only in vascular endothelial cells in the infarct area of a transient middle cerebral artery occlusion (tMCAO) model but not in other brain cells (Wang et al., 2020). With the extension of reperfusion time, LCN2 started to express in the astrocytes, neutrophils, and endothelial cells. This finding implies that vascular endothelial cells are an important source of LCN2 after ischemic-reperfusion injury and may act as an inhibitory target, preventing LCN2 elevation. Notably, the infarct volume, neurological deficits, immune infiltrates, and pro-inflammatory molecules (IL-1β, TNF-α, CCL2, and CXCL1) were attenuated in LCN2^–/–^ mice after ischemic stroke. On the contrary, LCN2 over-expressed mice exhibited larger infarct volumes and worse behavior scores than the control group. Moreover, intraperitoneal injection of LCN2 monoclonal antibody at 4 h after tMCAO significantly reduced brain injury (Wang et al., 2020).

Furthermore, LCN2 in endothelial cells significantly reduced the *trans*-endothelial electrical resistance (TEER), which was associated with BBB disruption (Ahn et al., 2020). Co-culture neurons with LCN2^+/+^ astrocytes exhibited more neurotoxicity than LCN2^–/–^ astrocytes on day 1 (Bi et al., 2013). Similar results were observed in another tMCAO model. Total infarct area, brain swelling volume, and neurological deficit scores were worse in the WT mice than in the LCN2 null group (Rathore et al., 2011). However, there were no differences in infarct volume, brain edema, or neurological scores between the WT and LCN2^–/–^ groups in a permanent MCAO (pMCAO) model with no reperfusion. These results suggest that LCN2 might be involved in the progress of reperfusion injury after ischemic stroke.

Numerous LCN2^+^ neutrophils were detected in the infarct hemisphere in the LCN2 positive vessels at 23 h after tMCAO, indicating that blood vessels are the major pipelines for LCN2^+^ neutrophils to infiltrate into injury sites after ischemic stroke. The immunolocalization results revealed that LCN2 was primarily expressed in the neutrophils, vascular endothelial cells, and astrocytes at 23 h after tMCAO. Wang et al. (2020) found the LCN2 levels in the brain were increased significantly at 1 h in WT mice after tMCAO compared to LCN2^–/–^ mice (Bokhari et al., 2014). LCN2 levels peaked at 23 h and then decreased by 48–72 h. In 1996, Elnelhoum et al. analyzed LCN2 levels in plasma from 156 patients between one day and three days after stroke (Elneihoum et al., 1996). They found that the expression of LCN2 was significantly raised in the stroke and TIA groups than in the control group. Furthermore, there is a clear link between LCN2 levels and leukocyte cell counts in the blood. The results demonstrated that elevated LCN2 is essential in early brain injury after ischemic stroke.

## LCN2 expression in hemorrhagic stroke

LCN2 also engages in the pathological progress of brain damage after a hemorrhagic stroke. Following hemorrhage in the ICH model, numerous LCN2 was found in the basal ganglia, primarily in astrocytes, microglia/macrophages, neutrophils, and endothelial cells (Dong et al., 2013). Overexpressed LCN2 in the injury hemisphere can last for more than 2 weeks. LCN2 concentrations reached about 80-fold higher levels in the ipsilateral hemisphere compared to the contralateral hemisphere on day 3 and decreased until the 14th day after ICH. LCN2 deficient mice demonstrated less microglial activation, brain swelling, neurological deficits, and brain atrophy after ICH (Ni et al., 2015).

Moreover, evidence indicated that LCN2 expression in the cerebrospinal fluid (CSF) is a reliable biochemical marker in diagnosing brain vascular diseases and relates to unfavorable prognosis (Wang, 2012; Kim et al., 2017; Llorens et al., 2020). The concentration of LCN2 in CSF and blood was significantly higher in SAH patients than in non-SAH patients (Yu et al., 2021). Furthermore, elevated LCN2 in CSF was associated with poor modified Rankin Scale (MRS) at 3 and 6 months. The author also found a positive relationship between LCN2 expression and the levels of IL-6. TNF-α and MMP9 in the CSF. Similarly, clinical research focuses on the role of LCN2 in aneurysmal SAH patients and found that overexpressed LCN2 in CSF is related to severe SAH scores. Another long-term research indicated the brain.

The studies that focus on the role of LCN2 in IVH are few. However, limited research depicted that LCN2 upgraded at about 20-fold levels compared to the saline control group at 24 h in the astrocytes, neurons, and microglia/macrophage in both cortex and periventricular area (Holden et al., 2014). Further results demonstrated that the enlargement volume of ventricular was significantly smaller in LCN2^–/–^ mice compared to WT mice.

## Regulation of LCN2 in brain after stroke

**FIGURE 3:**
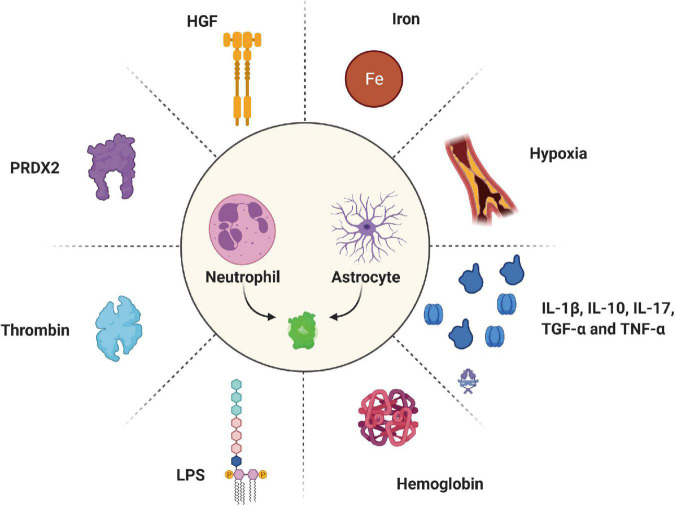
Various inducers regulate the expression of LCN2 under pathological conditions. HGF, hepatocyte growth factor; PRDX2, peroxiredoxin 2; LPS, lipopolysaccharides.

Many factors can induce LCN2 expression under pathological conditions (Figure 3). Hypoxia is significant pathological progress after ischemic stroke associated with poor outcomes and neurological deficits (Nathaniel et al., 2015; Ferdinand and Roffe, 2016). Large amounts of LCN2 were expressed on activated astrocytes around the infarct area for 2 h under hypoxic conditions. Hypoxia elevates LCN2 in brain astrocytes, the liver, serum, and other tissues (Jiang et al., 2008). Neutrophils mainly express the LCN2 in serum under hypoxic stimulation. These neutrophils can penetrate hypoxia brain tissue through the BBB to exert injury. Meanwhile, LCN2 can induce the expression of hypoxia-inducible factor 1α, a transcription regulator, and finally promote the secretion of IL-6 and IL-8 (Ke and Costa, 2006; Holden et al., 2014). Moreover, the study found that the IKKβ-mediated NK-κB signaling pathway activated in the SMCs after damage eventually upregulated the levels of LCN2 (Bu et al., 2006). In addition, people found that other inflammatory cytokines can induce LCN2 under pathological conditions, such as IL-10, IL-17, TGF-α, and TNF-α (Moschen et al., 2016; Yammine et al., 2019).

The lysates of red blood cells are the major factors that induce the expression of LCN2 after stroke. Hemoglobin, released from lysed red blood cells a few hours after a hemorrhagic stroke, was verified to induce LCN2 (Shishido et al., 2016). In the IVH model, high hemoglobin levels in the brain ventricular were associated with severe hydrocephalus and brain injury (Strahle et al., 2012; Chen et al., 2015). Compared to LCN2^–/–^ mice, an injection of hemoglobin into the brain ventricle of WT mice significantly increased LCN2 expression around the periventricular space. In a hemoglobin-induce IVH model, the author detected many LCN2 positive cells in the periventricular and cortical area after injecting 20 μL of hemoglobin. Double-labeling immunofluorescence depicted that LCN2 is mainly located in astrocytes. In addition, hemoglobin induced less ventricular volume enlargement and glial cell activation in LCN2 deficiency mice than in WT mice. However, the mechanism by which hemoglobin induces the LCN2 signaling pathway following IVH requires more studies.

Iron is another harmful factor released from hematoma during clot dissolution (Armengou and Davalos, 2002; Garton et al., 2016; Gill et al., 2018). A recent study revealed that an injection of FeCl_2_ (10 μL) into the basal ganglia induced the expression of LCN2 by nearly fivefold compared with the control group (Ni et al., 2015). An injection of 30 μL of iron caused more than a 130-fold increase in LCN2 levels on day 3 than the control group (Dong et al., 2013). In addition, iron-induced brain edema and BBB disruption vastly decreased after LCN2 deficiency (Ni et al., 2015). Meanwhile, LCN2, a small molecular weight siderophore, was thought to reduce iron concentrations released from the hematoma by transferring iron into intracellular space through 24p3R (Flo et al., 2004; Devireddy et al., 2005).

Notably, reducing iron overload after ICH can significantly down-regulate the expression levels of LCN2. The study also found that the level of LCN2 induced by ICH decreased after using deferoxamine, an iron chelator. These results revealed that LCN2 is vital in promoting brain damage by regulating iron overload after ICH.

Peroxiredoxin-2 (PRDX2) is the third most abundant protein in red blood cells and acts as an injury factor in brain injury after stroke (Bian et al., 2020; Tan et al., 2020). Our previous study found that an injection of recombinant PRDX2 (15 μL) into the caudate nucleus causes a large amount of LCN2. In addition, LCN2 was found to be expressed on neutrophils, astrocytes, and endothelial cells, eventually leading to brain damage in both male and female mice (Zhang et al., 2021). However, the signaling pathways of PRDX2-induced LCN2 expression remain unclear.

Another factor thought to be linked to LCN2 expression is thrombin. Increasing evidence clarified that LCN2 also contributes to thrombin-induced brain injury after ICH. In the ICH model, Elevated LCN2 induced by thrombin is related to brain swelling and neurological deficits. Mao et al. (2016) found that thrombin, at the injection of 0.4 U into basal ganglia, significantly induced LCN2 levels in WT mice than in the control group. This study also found that thrombin caused less LCN2 expression in the PAR-1^–/–^ (a receptor of thrombin) than WT mice, along with less brain edema, neuroinflammation, and neurological deficits. Moreover, these adverse effects were attenuated in LCN2^–/–^ compared to WT mice. LCN2^–/–^ mice with a con-injection of LCN2 and thrombin into the basal ganglia displayed more brain damage than the control group. These results suggested that thrombin can induce LCN2 through its receptor-PAR-1. The components from erythrocytes might be the principal causes for generating LCN2 after ICH. In turn, the high LCN2 levels can enhance their role in promoting brain injury.

Hepatocyte growth factor (HGF) was identified as another inducer upregulating LCN2 mRNA levels (Koh and Lee, 2015). The study indicated that HGF could mediate LCN2 expression in a dose-dependent manner. Lipopolysaccharide (LPS) also can promote the expression of LCN2 under neuroinflammation (Ip et al., 2011; Ondee et al., 2019). Jin et al. (2014a) found that LCN2 was strongly expressed in the brain after injection of LPS in WT mice on day 1. Furthermore, LPS-induced neuronal death and glial activation were reduced in LCN2 knockout mice. The study also found the protection value of LCN2 in LPS-induced inflammation. Compared with WT mice injected with LPS, LCN2^–/–^ mice demonstrated high levels of pro-inflammatory cytokines (TNF-α and IL-1β) in the brain and exhibited a significantly worsening behavioral phenotype (Kang et al., 2018).

In addition, studies found that the signaling pathway JAK/STAT participates in the expression of LCN2 by promoting the binding of Stat5 to the LCN2 promoter (Zhao and Stephens, 2013; Zhao et al., 2014). Furthermore, the HER/phosphoinositide 3-kinase/AKT/NF-κB pathway is another promotor associated with LCN2 expression (Wang et al., 2015a). In addition, LCN2 activates many downstream signaling pathways, such as NF-κB/STAT3, EGFR, TGF-β1/LCN2/Twist1 (Wang et al., 2013; Guo et al., 2014; Yammine et al., 2019). However, the specific mechanisms that induce the overexpression of LCN2 in various cells in the central nervous system after stroke require further study.

## LCN2 with neuroinflammation after stroke

**FIGURE 4:**
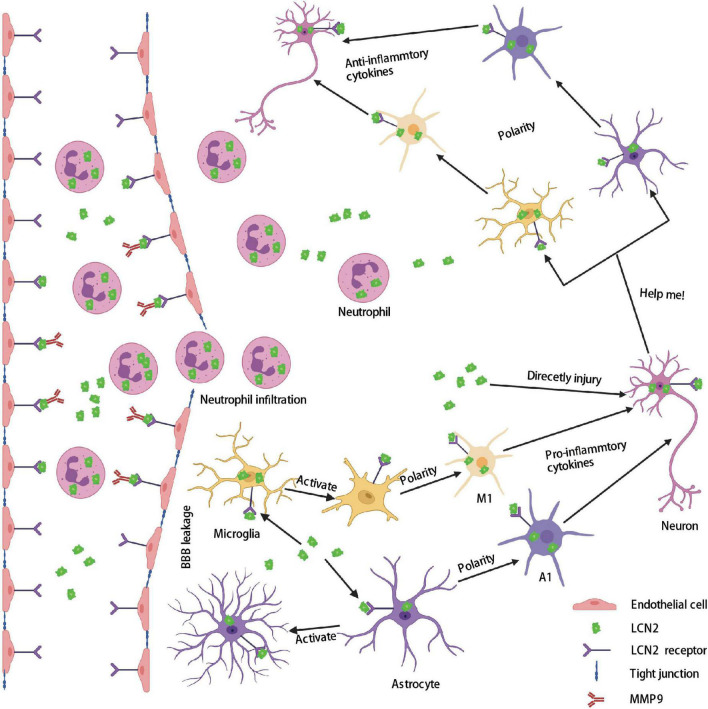
Role of LCN2 in the brain after stroke. Firstly, a large amount of LCN2 was up-regulated both in the peripheral blood and brain after stroke. Secondly, LCN2 activates MMP9 and forms a complex that binds to endothelial cells through the LCN2 receptor, and then destroys tight junctions to form BBB leakage. Thirdly, neutrophils enter the brain through BBB leakage. The highly expressed LCN2 in the brain activates microglia and astrocytes and polarizes them into a proinflammatory phenotype (M1 and A1), which directly or indirectly injury neurons. Lastly, damaged neurons release LCN2 as a help-me signal and polarizes microglia and astrocytes into an anti-inflammatory phenotype (M2 and A2). BBB, blood–brain barrier; LCN2, lipocalin 2; MMP9, matrix metallopeptidase 9.

Neuroinflammation, caused by damage-associated molecular patterns (DAMPs) following stroke, leads to secondary brain injury and poor prognosis (Jayaraj et al., 2019). Several pathological signs of progress contribute to neuroinflammation after stoke, including astrocytes and microglia/macrophages activation and polarization, neutrophils infiltration, pro-inflammatory cytokines generation, which finally induce brain edema, BBB disruption, neuronal death, and neurological deficits (Dabrowska et al., 2019; Jayaraj et al., 2019; Yang et al., 2019). Increasing evidence emphasizes the role of LCN2 in these signs of progress after stroke (Figure 4).

## LCN2 and astrocytes

The activation and polarization of astrocytes play an important role in brain injury after stroke. Based on different characteristics, reactive astrocytes are divided into helpful cell types (A2) and harmful cell types (A1) (Liddelow and Barres, 2017; Pivonkova and Anderova, 2017; Morita et al., 2019). A2 astrocytes can secret protective factors that promote neuronal survival and tissue repair, whereas A1 astrocytes cause rapid death of neurons and oligodendrocytes (Li et al., 2019). LCN2, secreted by activated astrocytes, has been shown to increase the conversion of activated astrocytes into pro-inflammatory types and directly injure surrounding neurons (Bi et al., 2013). LCN2 alters the phenotype of astrocytes in a positive feedback manner through a Rho-ROCK (Rho-kinase)-GFAP pathway under inflammatory conditions (Lee et al., 2009; Jang et al., 2013a). Astrocytes pre-treated with LCN2 exhibited activate phenotype by secreting classical activate markers, such as IL-1β, INOS, TNF-α, and CXCL10 (Lee et al., 2011). Further study indicated that LCN2 could negatively mediate the phosphorylation of the IL-4-induced STAT6 pathway (Garg et al., 2015).

Autocrine LCN2 from astrocytes also mediates the migration and death of astrocytes. In the IVH model, injection of recombinant LCN2 into WT mice significantly induced fewer glial fibrillary acidic protein (GFAP) positive cells than into LCN2^–/–^ mice. Our previous study indicated that recombinant LCN2 (10 μl) induced multiple GFAP^+^ cells in LCN2 deficient mice on day 1 after ICH. In another stimulated cerebral ischemic model, the author found that LCN2 increased the expression of astrocytes in a dose-dependent manner. LCN2 can promote the migration of astrocytes by upregulating CXCL10 through the JAK2/STAT3 and IKK/NK-κB pathways. CXCL10 expression and migration ability of astrocytes were suppressed in LCN2^–/–^ mice.

## LCN2 and microglia/macrophages

Microglia/macrophages, according to different makers, also can polarize into two categories: M1 microglia/macrophage (pro-inflammatory) and M2 microglia/macrophage (anti-inflammatory). In an LPS-induced neuroinflammatory model, Jang et al. (2013b) found that recombinant LCN2 remarkedly induced microglia/macrophages to polarize to M1 subtypes. Meanwhile, the M1-related genes in microglia/macrophages were suppressed in LCN2 deficient mice. In addition, the study found that LCN2 can inhibit the polarization of microglia/macrophages to M2 subtypes by negatively regulating the IL-4/STAT6 pathway (Sica and Bronte, 2007). Moreover, the expression of IBA-1^+^ microglia/macrophages and ARG1 (a classical marker of M2) were suppressed in the LCN2 deficient mice (Huang et al., 2016). Furthermore, the morphology of microglia changed from a normal ramified resting phenotype to a long-rod phenotype with fewer peripheral branches after treatment with LCN2 (Xing et al., 2014). Meanwhile, the length of microglia spread by more than 50% compared to the control group after LCN2 treatment for 1 day. In an LPS-induced bone-marrow-derived macrophages (BMDMs) model, the author found that recombinant LCN2 also suppressed IL-6, CD11c, and INOS (markers of M1) expression in LCN2^–/–^ BMDMs (Guo et al., 2014). In addition, LCN2 deficiency mice showed fewer levels of CD68 and CD86 (M1 markers) (Chun et al., 2015). However, the specific pathways behind the relationship between LCN2 and activation and polarization of glial cells are still far from uncovered.

## LCN2 and neutrophils

Neutrophils are the first infiltrating immune cells that induce BBB disruption, brain edema, and neuronal death after stroke (Jickling et al., 2015). In normal conditions, LCN2 but not 24p3R was enriched in neutrophils. LCN2, a composition of neutrophil secondary granules, is closely related to infiltrated neutrophils after stroke (Dahl et al., 2018). Our previous studies found that neutrophils in LCN2^–/–^ mice significantly decreased on day 1 after ICH, which ultimately reduces brain injury. An injection of 10 μl recombinant LCN2 can increase MPO levels by nearly three times in the LCN2^–/–^ mice compared to the saline control group on day 1 (Mao et al., 2016). PKCδ expresses in the neutrophils and is essential in promoting reperfusion damage after ischemic stroke (Parihar et al., 2018). Weng et al. (2015) identified PKCδ as an initiating factor that promotes neutrophils to release LCN2 and enter the injury sites. In-depth research found that LCN2 activates Erk1/2 pathways by binding to 24p3R, ultimately regulating neutrophils’ participation in inflammation (Zhao Y. et al., 2019). LCN2 also activates the MEK1/2 and MAPK pathways through the 24p3R receptor on neutrophils to produce pro-inflammatory cytokines, such as IL-1α, IL-6, IL-8, and TNF-α (Shao et al., 2016). Neutrophils from LCN2^–/–^ mice exhibited lower CD11b, CD51, and CD62L (important adhesion molecules of neutrophils) expression, which demonstrated a significantly lower adhesion capacity than the neutrophils from WT mice. Furthermore, the chemokine receptor CXCR2 was suppressed in LCN2 deficient neutrophils, making neutrophils less susceptible to the chemotactic response after neuroinflammation (Schroll et al., 2012). In summary, these data indicated that LCN2 not only induces neutrophil infiltration but also regulates the function of neutrophils under neuroinflammation conditions.

## LCN2 and neurons

Our previous study discovered that LCN2 and its receptor-24p3R were expressed in neurons after stroke. Chen X. et al. (2020) found that LCN2 and LCN2R in rat hippocampal neurons increased significantly after injury and induced neuronal apoptosis in a mitochondrion-related manner. Xing et al. (2014) concluded that released LCN2 from injured neurons would send out a “help-me” signal, thereby promoting glial cells to polarize to pro-recovery phenotypes that ultimately lead to beneficial results after ischemic stroke.

## LCN2 and other cells

LCN2 participates in the homeostasis and migration process of SMCs (Tarin et al., 2016; Wang et al., 2017). However, the actual role of LCN2 in endothelial cells remains complex and ambiguous. Some studies found that LCN2 can promote angiogenesis through iron and reactive oxygen-related pathways (Wu et al., 2015). LCN2 may also act as an endogenous “help-me” signal that reduces endothelial permeability by restoring the membrane distribution of the tight junction protein ZO-1 and the adhesion junction protein VE-cadherin (Du et al., 2019). However, the researchers focus on the liver-brain axis and found that increased circulatory LCN2 can promote BBB dysfunction by influencing the expression of tight junction protein Claudin 5 and the secretion of pro-inflammatory cytokines (IL-6 and IL-1β). Angiogenesis is crucial to recovery progress after a stroke (Mondal et al., 2020). Wu et al. (2015) found that LCN2 can induce matrigel tube formation and scratch migration in a reactive oxygen species-dependent and iron-dependent manner, ultimately promoting angiogenesis.

## LCN2 contributes to white matter injury and thrombosis after stroke

Whiter matter injury (WMI) is a brain injury in preterm infants, with an incidence of up to 50% in very low birth weight infants (Volpe, 2003; Dyet et al., 2006). The study found that LCN2 contributed to the acute WMI after SAH. LCN2 positive cells were detected in the damaged white matter, which was co-expressed with glial cells (Egashira et al., 2014). LCN2^–/–^ mice displayed less axonal damage and myelin degradation in white matter than in the WT group. Meanwhile, there has a close relationship between LCN2 expression and BBB leakage after SAH. Egashira et al. (2014) found many LCN2 positive cells in the corpus callosum on day 1 after SAH, contributing to BBB disruption (Garg et al., 2015). Furthermore, less volume of MRI T2-hyperintensity was observed in LCN2 deficient mice than in WT mice on day 1. Immunohistochemistry showed that 24p3R is mainly expressed on oligodendrocytes, astrocytes, endothelial cells, and pericytes in the white matter. These results show that LCN2/24p3R signaling might be an essential factor in WMI after SAH.

Cerebral vascular thrombosis is associated with an adverse outcome in patients after SAH (Kato et al., 2010; Sharma et al., 2010). In addition, recent research detected the formation of early brain vessel thrombosis 4 h after SAH using MRI (Wang Z. et al., 2021). The author found that LCN2 deficiency mice have fewer T2*-positive vessels, lower SAH grade, less T2 lesion, BBB leakage, and neuronal death. The exact mechanism of LCN2 leading to early brain vessel thrombosis after SAH remains unclear. This study identifies a promising target for improving early brain injury following SAH.

## LCN2 contributes to blood–brain barrier dysfunction

Matrix metalloproteinase 9 (MMP9), which increased significantly after stroke, is well known as a critical mediator that induces BBB dysfunction and brain edema (Egashira et al., 2015; Turner and Sharp, 2016). MMP9 can destroy the extracellular matrix (ECM), basal lamina, and tight junction proteins necessary to maintain the BBB’s normal construction. Increased evidence found that LCN2 can activate MMP9 by directly binding to MMP9, which finally destroys the integrity of BBB after stroke (Yndestad et al., 2009; Chou et al., 2015). Studies found that serum LCN2/MMP9 complex levels were higher in hemorrhagic stroke patients than in control groups and were a suitable predictive marker for poor prognosis (Feiler et al., 2011; Phillips et al., 2014). Furthermore, intracerebral injection of recombinant LCN2 results in more brain swelling and albumin immunoactivity than in the saline group, alleviated after LCN2 deficiency (Toyota et al., 2019).

## LCN2 and iron

It is well-known that iron is essential for many physiological processes in the body, including carrying oxygen, synthesis enzymes, transporting electrons, and stabilizing deoxyribonucleic acid (DNA) (Abbaspour et al., 2014; Waldvogel-Abramowski et al., 2014). LCN2 was first described as an antibacterial siderophore-binding protein that prevents microbes from absorbing iron, which is critical for iron depletion in the innate immune system (Goetz et al., 2002).

## LCN2 regulate iron homeostasis

Iron overload after hemorrhagic stroke is unfavorable and predicts an adverse outcome. Iron overload induces brain injury *via* many signaling pathways, such as creating free radicals and promoting ferroptosis (Siesjo et al., 1989; Doll and Conrad, 2017). In addition, studies indicated that iron overload after hemorrhagic stroke contributes to acute brain edema, neuronal toxicity, ventricular enlargement, and delayed brain atrophy (Wu et al., 2003; Gu et al., 2009; Chen et al., 2011).

LCN2 regulates brain iron homeostasis under normal and pathological conditions. LCN2 in the normal brain helps transport iron into the neuron through the receptor of 24p3R. Meanwhile, increased LCN2 after neuronal injury or neuroinflammation might improve the remarkable ability to mediate iron homeostasis (Chia et al., 2011). Xiao et al. (2016) detected an enormous amount of LCN2 in a con-culture of bone marrow macrophages with ferrous iron. This result means macrophages can secrete LCN2 under inflammatory conditions to maintain an iron balance. Astrocytes and microglia were found to enrich high iron levels under inflammatory conditions (Rathore et al., 2012; Pelizzoni et al., 2013; Holland et al., 2018). The study found that this iron was not bound with transferrin, which LCN2 might regulate. The iron-binding form of LCN2 can increase the mobility of the neuronal dendritic spine, spread the mushroom spines, and promote spine maturation (Mucha et al., 2011). Dekens et al. (2018) detected that LCN2^–/–^ mice had higher iron intensity in hippocampal subregions than WT mice.

Elevated LCN2 is also related to iron overload after a hemorrhagic stroke. The author found fewer ferritin-positive cells in LCN2^–/–^ mice compared to WT mice on day 1 in the ipsilateral basal ganglia after ICH (Ni et al., 2015). Meanwhile, the expression of FTH and FTL proteins (ferritin subunits) was lower in LCN2^–/–^ mice. Ferrous iron significantly increased LCN2 density in WT mice compared to the saline group. In addition, MRI T2 lesion, brain edema, and BBB leakage were attenuated in LCN2^–/–^ mice than in WT mice after injection of ferrous iron on day 1. The ICH-induced LCN2 levels were down-regulated after using deferoxamine, an iron chelator, which eventually caused minor brain injury (Dong et al., 2013).

## LCN2 mediate ferroptosis

Ferroptosis, an iron-dependent, non-apoptotic mode of cell death characterized by the accumulation of lipid reactive oxygen species (ROS), was first described by Dixonin 2012 (Dixon et al., 2012). After the stroke, ferroptosis drew increasing attention as a new type of cell death. A recent study found that overexpressed LCN2 inhibits ferroptosis cell death *via* the NUPR1-LCN2 pathway and ultimately reduces iron accumulation and subsequent oxidative injury (Liu et al., 2021). Catalytic iron (CI) is a promoter that induces cell death in the form of ferroptosis (Dixon et al., 2012). Xiao et al. (2016) found that LCN2 can alleviate iron toxicity by reducing catalytic iron generation and preventing iron-mediated ferroptosis. We speculate that LCN2 may play a more significant role in ferroptosis as a critical factor in maintaining iron homeostasis, and its specific mechanisms merit further investigation.

## Protective role of LCN2 after stroke

Phagocytosis is essential for the clearance of apoptotic cells, extracellular protein aggregates, and infectious bacteria by microglia in the central nervous system, and is one of the important mechanisms by which they exert neuroprotective effects (Lian et al., 2016). LCN2 was found to act as a beneficial factor by sending a “help me” signal after an ischemic stroke (Xing et al., 2014). The author found that LCN2 released from the damaged neurons can activate and enhance the microglia’s phagocytic ability, finally promoting recovery. In addition, it has been proposed that IL-10 is an anti-inflammatory factor that can promote the recovery of injured neurons (Zhou et al., 2009). After using recombinant LCN2, the concentrations of IL-10 released by microglia increased significantly, while the expression of INOS and CD206 remained unchanged. Similarly, recombinant LCN2 significantly increased the expression of GFAP, along with the levels of TSP-1 and BNDF, which were thought to provide neuroprotection (Norrix et al., 2012; Miranda et al., 2019). Furthermore, the author discovered that LCN2-activated glial cells promote neuroplasticity by inducing the expression of synapse binding protein. Meanwhile, in an infection model of IL-10-deficient mice, LCN2 was shown to prevent spontaneous colitis by enhancing phagocytic bacterial clearance by macrophages (Toyonaga et al., 2016).

Angiogenesis, which occurs after stroke, restores blood supply and provides oxygen and nutrients to ischemic brain tissue, a process thought to facilitate stroke recovery (Adamczak and Hoehn, 2015). LCN2 was also found to promote the angiogenesis of endothelial cells (Wu et al., 2015). The migration and matrigel tube formation of endothelial cells was significantly enhanced after treatment with LCN2 on day 2. LCN2 was found to induce chemokine production, promote macrophage migration and phagocytosis, and mediate neutrophil migration and chemotaxis in inflammatory responses (Wang et al., 2019). A recent study demonstrated that LCN2 was able to reduce LPS-induced inflammation by altering macrophage properties (Du et al., 2021). Transcripts of pro-inflammatory cytokines were significantly increased in bone marrow-derived macrophage (BMDM) from LCN2^–/–^ mice, whereas transcripts of anti-inflammatory cytokines were significantly decreased compared with cytokines in BMDM from WT mice. Persistent activation of STAT1, STAT3, and JNK pathways was found in LCN2^–/–^ mice, accompanied by increased inflammatory cytokines and chemokines (IL-1β, IL-6, TNF-α, and MCP-1/CCL2) increased expression (Borkham-Kamphorst et al., 2013). In addition, Asimakopoulou et al. (2016) found that LCN2 null mice exhibited less infiltration of neutrophils and leukocytes than WT mice. This result revealed the beneficial role of LCN2 in neuroinflammation. LCN2 may exert neuroprotective effects by affecting the expression of inflammatory cells and inflammatory factors after stroke. TNF-α is a significant risk factor after ischemic stroke (Bokhari et al., 2014). LCN2 shows a protective role on endothelial cells in TNF-α-induced brain injury (Du et al., 2019). Results indicated that LCN2 could significantly induce the expression of tight junction proteins (VE-cadherin and ZO-1), which eventually helps maintain the integrity of BBB.

Autophagy is a physiological mechanism that promotes energy cycling and orderly degradation through the self-regulatory breakdown of cellular components, helping to maintain homeostasis. A range of evidence suggests that autophagy is activated in response to stroke and has been well characterized as a therapeutic target. Appropriate autophagy after stroke can remove necrotic material against ischemic injury and promote neuronal survival (Mo et al., 2020; Wang X. et al., 2021). In an *in vitro* hypoxia model, Shujuan Qiu et al. found that LCN2 exerted a protective effect by activating autophagy and attenuating apoptosis through HIF1α (Qiu et al., 2018). Furthermore, LCN2 can also regulate apoptosis by interacting with IL-3 and IL-8 (Lai et al., 2013). GO analysis and KEGG pathway enrichment analysis revealed that 14 neuronal autophagy-related proteins were differentially expressed in LCN2^–/–^ mice compared with WT mice (Wu et al., 2021). For example, apoptosis-related proteins (such as apoptosis regulator BAX and DEP domain-containing mTOR-interacting protein) were significantly up-regulated, and seven apoptosis-related proteins (such as signal transducer and activator of transcription 1 and Ribosomal protein S6 kinase alpha-1) were significantly down-regulated. However, the studies focus on the protective effect of LCN2 after stroke are few until now, the specific signaling pathways involved in LCN2’s beneficial effects remain unclear.

## Targeting LCN2 for stroke therapy

**FIGURE 5:**
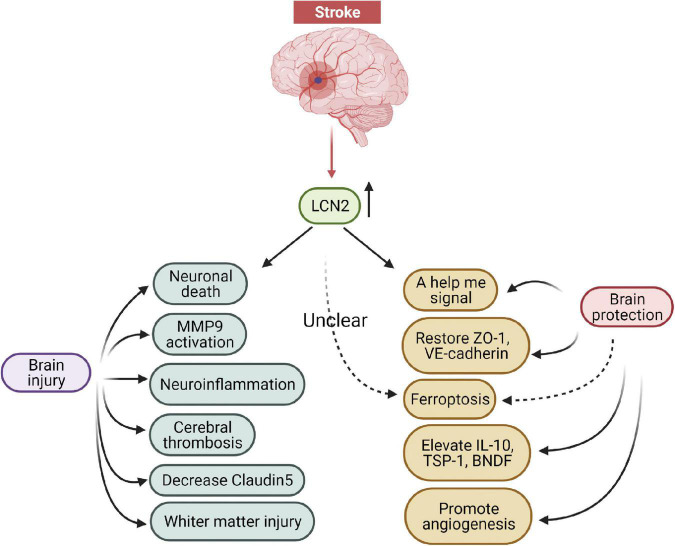
The damage and protection mechanisms of LCN2 in the brain after stroke.

Increasing studies have revealed that LCN2 was an essential mediator of brain injury and a promising therapeutic target following stroke (Figure 5). LCN2 deficient experimental animals alleviated immune infiltrates, neuronal death, BBB disruption, and neurological deficits under stroke conditions. In addition, specific antibodies blocking LCN2 similarly depicted neuronal protection after stroke. These findings support the possibility of LCN2 being used as a potential drug target in the future. Currently, several measures can be considered to reduce LCN2 mediated brain damage after stroke: (1) targeting LCN2 gene expression with gene editing or RNA interfering technologies; (2) inhibiting LCN2 protein elevation with specific antibodies; (3) binding LCN2 receptors with antagonists; (4) blocking LCN2 brain injury-related pathways; (5) activating LCN2 neuronal protection-related pathways. Among these approaches, exploring novel molecules that target LCN2-related signaling pathways (including damage and protection) appears to be the most suitable method for translational medicine needs in the future.

However, discovering small molecular drugs or inhibitors remains challenging because LCN2 is a non-enzymatic protein. Moreover, the mechanisms LCN2 engaged in brain injury are complex and ambiguous until right now. The current clinical trials focusing on LCN2 are limited to endocrine, urinary, and cardiovascular systems. As a result, future research should focus on the mechanisms underlying LCN2’s beneficial and harmful effects in the context of stroke and brain injury.

## Conclusion

LCN2 is an acute-phase protein released in large quantities after an ischemic and hemorrhagic stroke. LCN2 mainly expresses neutrophils, astrocytes, microglia/macrophages, endothelial cells, and neurons in the brain after stroke. Hypoxic conditions, thrombin, PRDX2, and iron contribute to the increase of LCN2 after stroke. High expression of LCN2 was associated with brain edema, BBB leakage, immune cell infiltration, glial cell activation and polarization, neuronal death, hydrocephalus, and neurological deficits in stroke models. Previous research has found that LCN2 released from injured neurons may act as a “help-me” signal, inducing a beneficial outcome. Upregulated levels of LCN2 in plasma and CSF predict an unfavorable prognosis in stroke patients. LCN2, along with its receptors 24p3R and megalin, plays an irreplaceable role in maintaining iron homeostasis and ferroptosis. So far, little is known about the signaling pathways that mediate the expression and function of LCN2 in stroke. Further studies on LCN2 and its receptors as promising therapeutic targets are required, especially in IVH and SAH. The relationship between LCN2 and tight junction proteins is complex and ambiguous, necessitating additional research in the future. Meanwhile, the specific process of the LCN2 and receptors on different brain cells needs to be explained in detail.

Furthermore, future research should focus on the upstream and downstream regulatory mechanisms of the LCN2 gene and protein, such as gene methylation modification, non-coding RNA regulation, protein phosphorylation, ubiquitination modification, and so on. These modifications determine the level of gene expression and protein activity after stroke. LCN2 not only plays a role in damage in stroke but also has a partial protective effect. Especially recently, it has been found that it may play an essential role in reducing ferroptosis. The specific mechanism behind it needs to be further explored. To sum up, fully understand the exact position of LCN2 and its receptors may benefit the prognosis of patients with stroke.

## Author contributions

JZ wrote the manuscript and drew figures and tables. ZW helped to modify the tables. HZ provided important guidance in the writing and revision of the manuscript. SL and JL provided overall supervision and edited the manuscript. HL and QC read and critically revised the manuscript. All authors contributed to the article and approved the submitted version.
